# Early Intervention With Obturators: A Case Series of Infants With Cleft Palate

**DOI:** 10.7759/cureus.79092

**Published:** 2025-02-16

**Authors:** Mrunal Pawar, Neha R Ghongade, Harshvardhan A Mohite, N.D. Shashikiran, Namrata Gaonkar

**Affiliations:** 1 Department of Pediatric Dentistry, School of Dental Sciences, Krishna Vishwa Vidyapeeth (Deemed to be University), Karad, IND; 2 Department of Pedodontics and Preventive Dentistry, School of Dental Sciences, Krishna Vishwa Vidyapeeth (Deemed to be University), Karad, IND; 3 Department of Pediatric and Preventive Dentistry, School of Dental Sciences, Krishna Vishwa Vidyapeeth (Deemed to be University), Karad, IND; 4 Department of Pedodontics and Preventive Dentistry, Krishna Vishwa Vidyapeeth (Deemed to be University), Karad, IND

**Keywords:** cleft palate, early intervention, feeding difficulties, infant, obturator, prosthetic device

## Abstract

A cleft palate is a congenital anomaly that can significantly impact an infant’s ability to feed and grow normally. This article presents a case series of three infants diagnosed with a congenital cleft palate, who were fitted with a custom obturator for feeding purposes. The infants exhibited difficulty with suction and oral feeding due to the cleft, which led to inadequate nutrition and increased risk of aspiration. To address this issue, a removable obturator was fabricated to temporarily occlude the palatal gap and improve the efficiency of sucking after taking consent for the same from the parents. The obturator was designed to ensure a secure fit, promote proper alignment, and reduce the risk of nasal regurgitation during feeding. The infants demonstrated immediate improvements in feeding and a significant reduction in aspiration risk. The article highlights the importance of early intervention and the role of prosthetic devices like obturators in managing feeding difficulties associated with cleft palate in neonates. Additionally, this report emphasizes the importance of a multidisciplinary approach involving pediatricians, speech therapists, and prosthodontists in the management of infants with cleft palate.

## Introduction

Cleft lip and palate (CLP) is a congenital condition influenced by both genetic and environmental factors. It occurs in approximately one in 750 live births, or 0.133% of the population [1]. Males are more likely to be affected, with the incidence being about twice as high in male children compared to females. Although CLP is often seen in families, with certain racial or ethnic groups being more predisposed, the condition can still occur in individuals without a family history [2]. Environmental factors, such as maternal health, nutrition, or exposure to teratogens, as well as new genetic mutations, can also contribute to its development. Thus, the absence of a family history does not rule out the possibility of CLP in an individual [3].

Infants born with this condition often experience significant feeding difficulties due to the inability to create the necessary suction for effective breastfeeding or bottle feeding. Direct breastfeeding is avoided most of the time due to fear of nasal regurgitation and bottle feeding or palatal spoon feeding is mostly encouraged during this period. Difficulties in feeding may compromise normal growth and disrupt the bonding process as breastfeeding helps in the development of oral musculature [3]. In addition, the gap between the oral and nasal cavities may cause milk to enter the nasal passages, increasing the risk of aspiration and compromising the infant's nutritional intake [4].

In the early days of life, surgical repair may not be immediately feasible, making it crucial to find alternative solutions to support feeding and prevent complications. Various strategies available include feeding bottles with wide openings, nasogastric tubes, palatal spoons, etc. [5]. One such solution is the use of an obturator, a removable prosthetic device designed to temporarily seal the palatal defect. This device helps improve the infant’s ability to suck by restoring normal oral function, while also preventing milk from entering the nasal cavity. The obturator serves as an important tool in managing feeding challenges, enabling adequate nutrition, and reducing the risk of aspiration until surgical intervention can be performed [5].

This article presents a case series of cleft palate patients who were provided with custom-made obturators. It details the process of fabricating these obturators, their role in enhancing feeding mechanics, and the positive outcomes in terms of improved feeding efficiency and safety. The series emphasizes the importance of early intervention with prosthetic devices as part of a multidisciplinary approach to cleft palate management in neonates, ensuring both nutritional support and the prevention of feeding-related complications.

## Case presentation

Case 1

**Figure 1:**
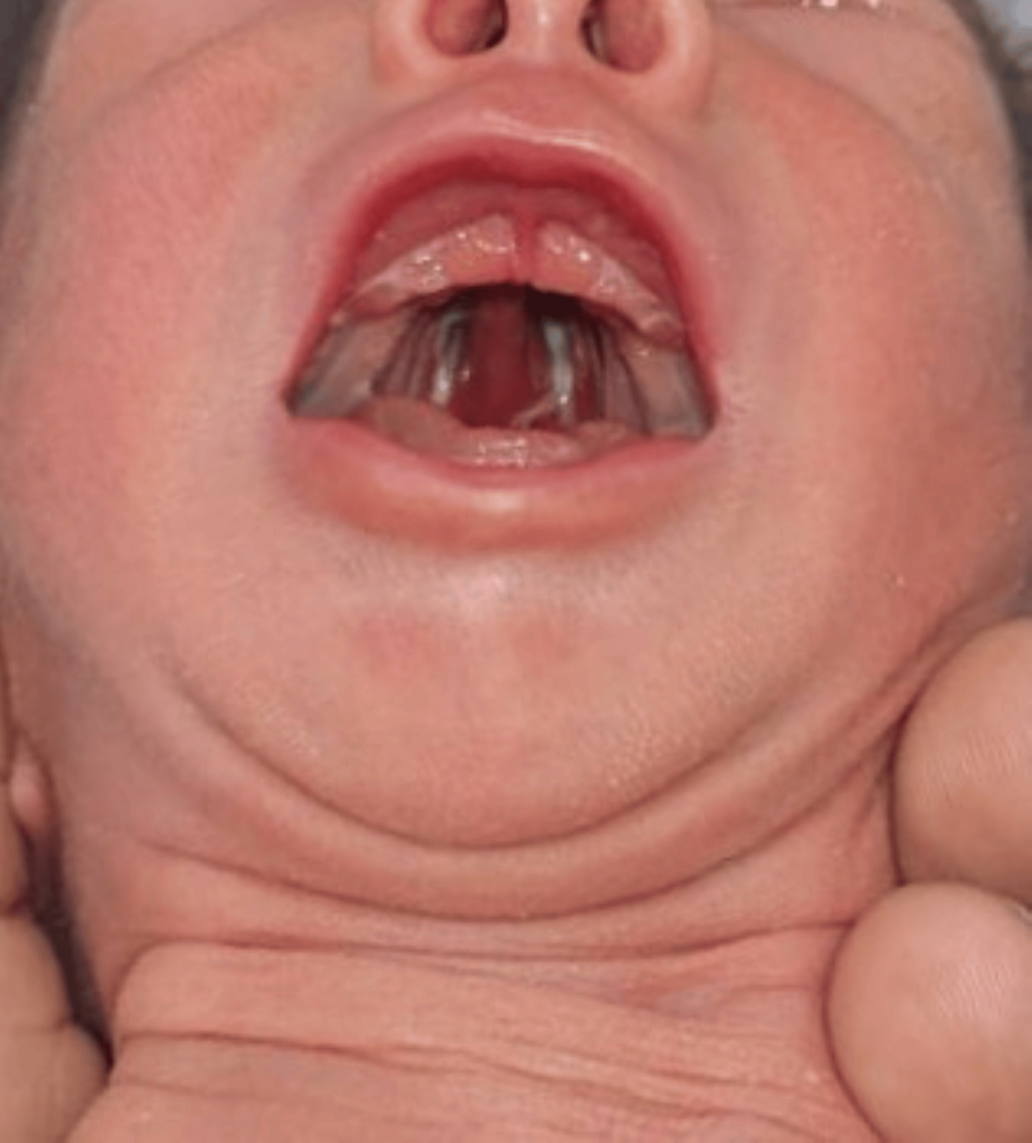
Intraoral view of the cleft present in the anterior palate region.

A three-day-old male neonate with the presence of a cleft in the hard palate (Figure 1) was referred to the Outpatient Department of the Pediatric and Preventive Dentistry, School of Dental Sciences, Krishna Vishwa Vidyapeeth, Karad, Maharashtra, India. The infant was born at full term via vaginal delivery, with an uneventful prenatal and perinatal history. There was no family history indicating cleft lip or palate or the use of teratogenic drugs but the parents gave a history of consanguineous marriage, which might have been a cause for the defect due to its high association as a risk factor for the presence of cleft in their children. However, upon clinical examination, a complete cleft palate involving a hard palate was noted. The cleft was characterized by a substantial gap in the palatal region, which was observed to extend from the anterior portion of the hard palate.

The infant exhibited marked difficulty in establishing proper suction for breastfeeding, as well as frequent nasal regurgitation of milk, which raised concerns about aspiration risk. The attending pediatrician noted that the baby was unable to effectively latch and feed, resulting in inadequate nutrition and poor weight gain, which warranted immediate intervention to address the feeding challenges. Upon examination, no other associated anomalies were found. The neonate was being fed via nasogastric intubation and had been fed only an hour prior. Consequently, the neonate was scheduled for impressions the following day. The parents were advised not to feed the baby for at least two hours before the impression-making procedure to prevent regurgitation and aspiration. A stepwise procedure for fabricating the palatal obturator is outlined below.

**Figure 2:**
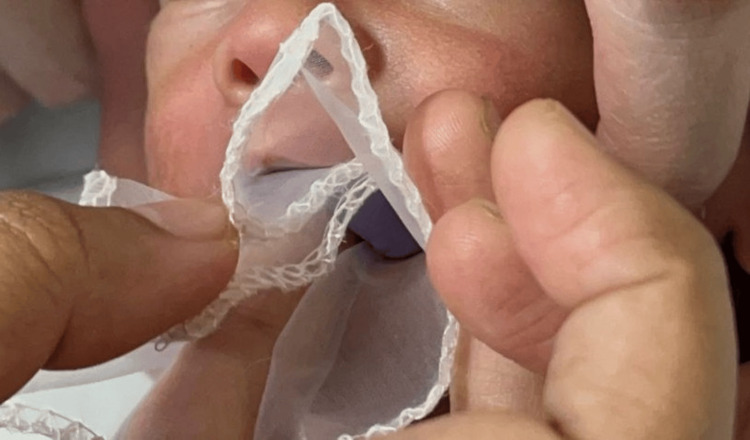
Maxillary impression making using putty impression material wrapped along with gauze.

The maxillary impression was made by the pediatric dentist when the infant was fully awake, without any premedication or anesthesia. The infant was positioned upright with the face forward position while impression making. Due to the small size of the infant's mouth, an impression tray or flat wooden stick could not be used as a carrier for the material. Therefore, it was decided to use finger-loaded impression material. Fast-setting elastomeric putty impression material was mixed and applied to the index finger. A large piece of gauze was wrapped around the loaded impression material, with the loose ends secured toward the hand (Figure 2).

**Figure 3:**
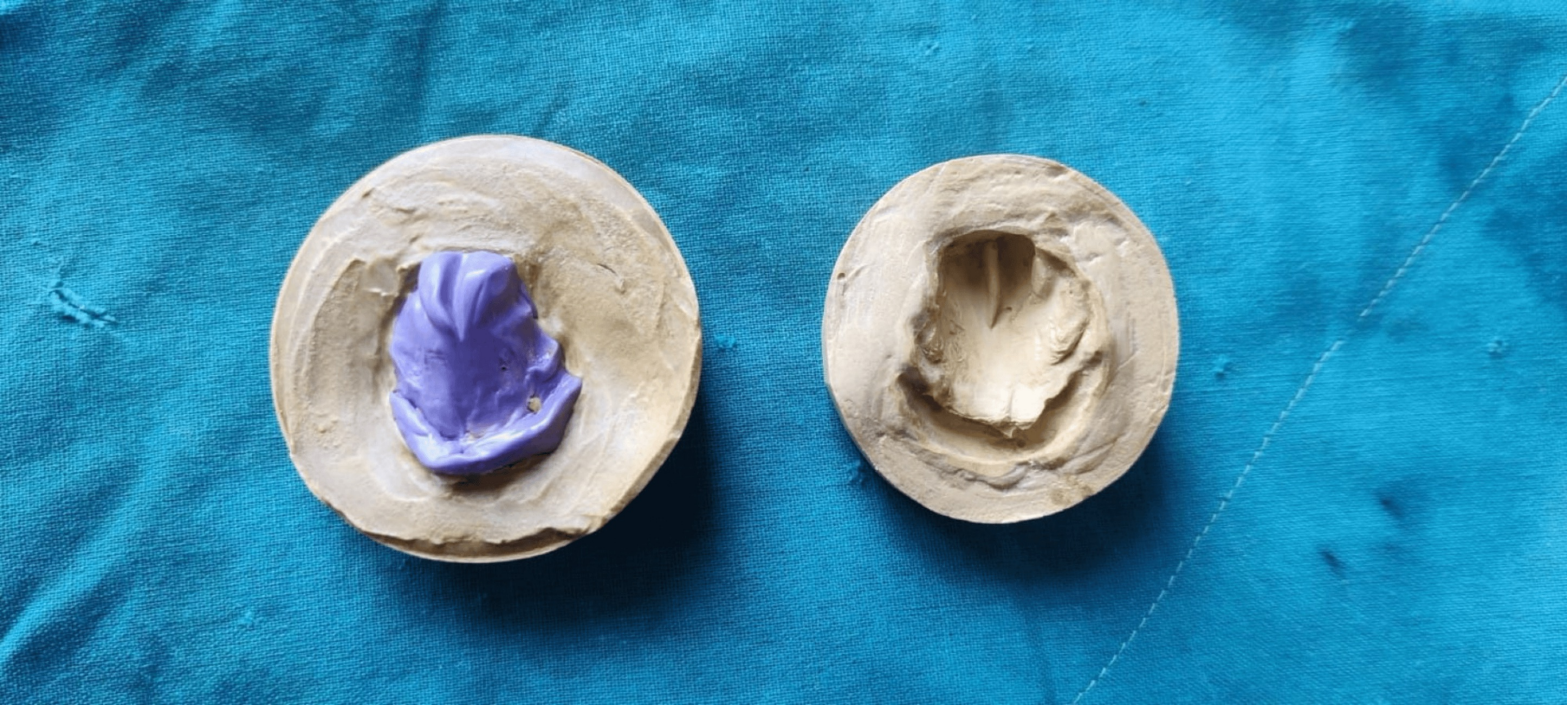
A putty impression was made and the master cast was retrieved using the inversion method.

The gauze helped control the material and prevented excessive displacement into the nasal cavity and pharynx. The thick consistency of the impression material prevented leakage through the gauze, keeping it contained within its flexible confines. The infant was encouraged to suck during the impression process, which aided in capturing tissue details and undercuts. Once the impression material was set, it was carefully removed from the infant’s mouth. The impression was then poured with dental stone, and the master cast was created using the inversion method (Figure 3).

**Figure 4:**
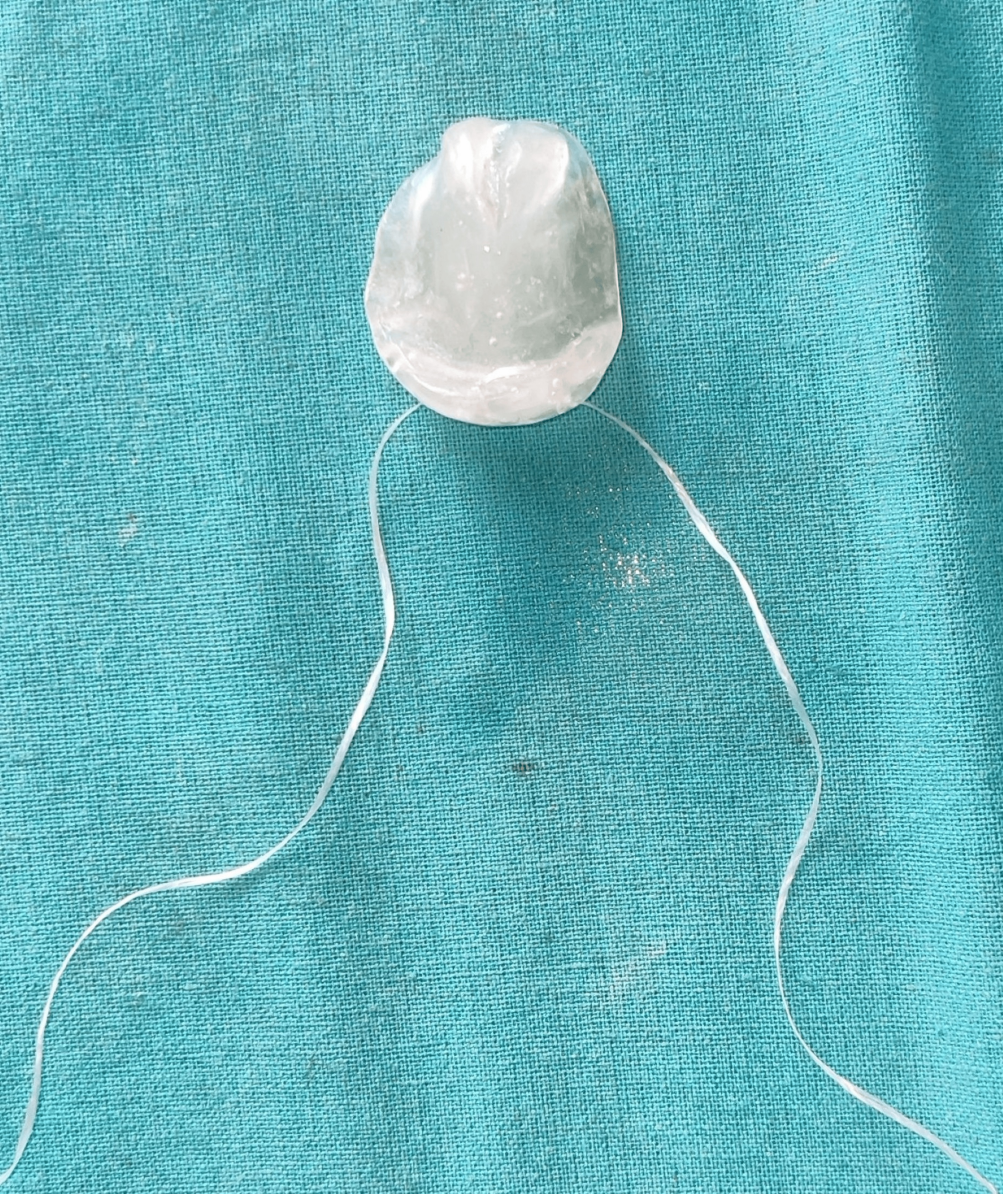
Feeding obturator with the safety thread attached.

A single-visit feeding obturator was fabricated using clear, flexible thermal-forming material with an extraoral midline extension for a hole to secure a safety thread (Figure 4).

**Figure 5:**
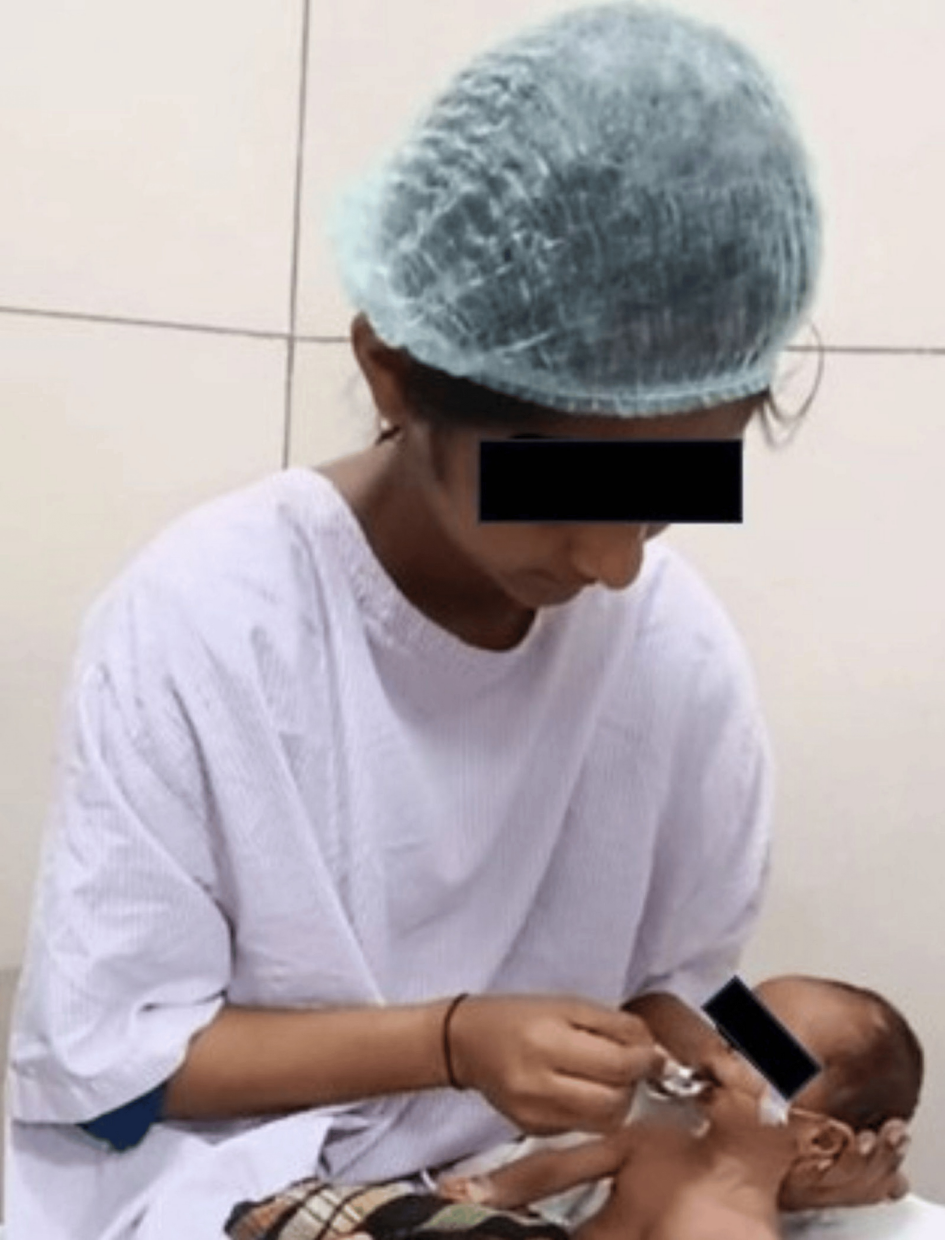
Training session for the mother.

The obturator was finished and polished to ensure smooth surfaces. After insertion, the neonate could comfortably suck with the feeding obturator. The mother was able to feed the child immediately without any nasal regurgitation after proper training and counseling sessions. She was properly trained about the attachment of safety threads and was told the importance of those threads for preventing aspiration (Figure 5).

The infant’s parents were educated about the process, and a follow-up appointment was scheduled. The case highlights the importance of early, non-surgical interventions in the management of feeding difficulties in neonates with cleft palate, ensuring optimal nutrition and preventing aspiration while awaiting definitive surgical correction.

Case 2

A two-day-old female neonate with the presence of a cleft in the hard palate was referred to the Outpatient Department of the Pediatric and Preventive Dentistry, School of Dental Sciences, Krishna Vishwa Vidyapeeth, Karad, Maharashtra, India. The prenatal and perinatal periods were uncomplicated, and the baby was delivered vaginally at full term. There was no related medical or family history of cleft lip or palate or teratogenic drug use. On clinical inspection, however, a full cleft palate affecting the hard palate was observed in the anterior region. Concerns regarding aspiration risk were raised by the infant's frequent nasal regurgitation of milk and noticeable difficulties establishing appropriate suction during breastfeeding. As a result, the feeding issues needed to be addressed right away. To avoid aspiration and regurgitation, the parents were instructed to refrain from feeding the infant for at least two hours before the impression procedure. Below is a detailed process for creating the palatal obturator.

**Figure 6:**
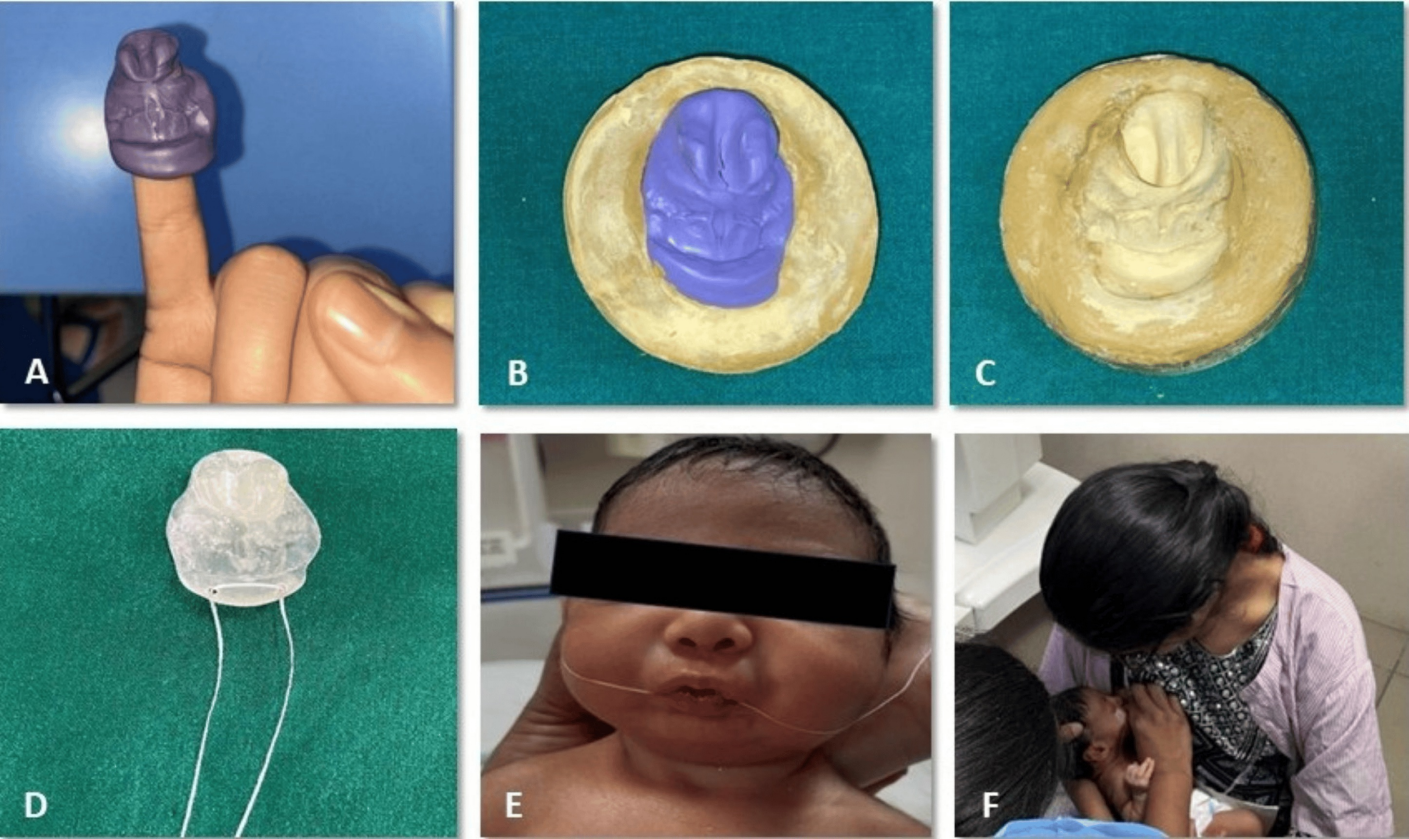
Stepwise procedure for fabrication of the obturator. A: Impression made using the finger. B: Impression mounted by inversion method. C: Retrieved master cast. D: Obturator fabricated along with the safety thread attached. E: Obturator in situ. F: Breastfeeding training for the mother after inserting the obturator to fill the defect.

During the impression-taking process, the baby was placed upright with their face front. The finger was imprinted with a mixture of fast-setting elastomeric putty. To ensure that the impression material did not extend toward the soft palate, the finger with the material was carefully inserted into the oral cavity. During the impression procedure, the baby was encouraged to suck, which helped to capture undercuts and tissue features. The impression material was gently taken out of the baby's mouth after it had set (Figure 6A). Dental stone was then poured into the impression, and the inversion procedure was used to generate the master cast (Figure 6B).

Using transparent, flexible thermal-forming material, a single-visit feeding obturator was created with an extraoral midline extension for a hole to fasten a safety thread. To guarantee smooth surfaces, the obturator was finished and polished (Figure 6C).

The newborn was able to use the feeding obturator to suck comfortably after it was inserted (Figure 6D). The mother received guidance on how to breastfeed while the baby was wearing an obturator (Figure 6E). The parents were instructed on how to maintain the oral hygiene of the baby after every meal. Additionally, they received instructions on how to keep the obturator clean.

A follow-up visit was set up. To ensure good nutrition and prevent aspiration while waiting for definitive surgical correction, the case emphasizes the significance of early, non-surgical measures in the management of feeding issues in neonates with cleft palate.

Case 3

A six-month-old male child with the presence of a cleft in the soft palate was referred to the Outpatient Department of the Pediatric and Preventive Dentistry, School of Dental Sciences, Krishna Vishwa Vidyapeeth, Karad, Maharashtra, India. The child was born at full term via vaginal delivery, with an uneventful prenatal and perinatal history. There was no family history indicating cleft lip or palate or the use of teratogenic drugs. However, upon clinical examination, a cleft of the soft palate was noted. The cleft was characterized by a gap in the posterior palatal region, which was observed to extend from the junction of the hard palate to the end soft palate.

The child demonstrated significant difficulty with establishing proper suction for breastfeeding, along with frequent nasal milk regurgitation, raising concerns about potential aspiration. Upon examination, no other abnormalities were detected.

**Figure 7:**
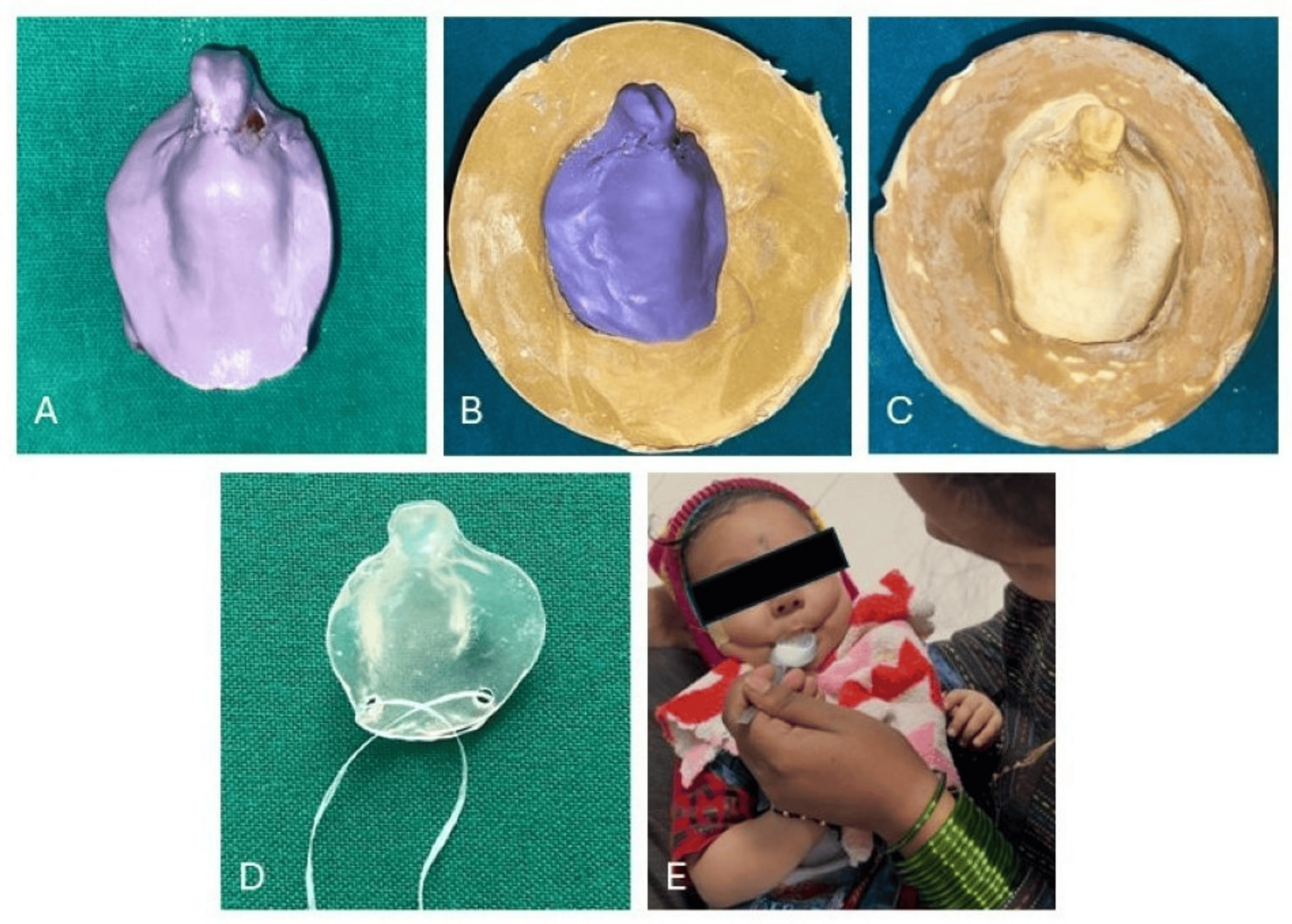
Stepwise procedure for fabrication of the obturator. A: Impression made using putty material. B: Impression mounted by inversion method. C: Retrieved master cast. D: Obturator fabricated with the safety thread attached. E: Obturator in situ and the mother trained for feeding the baby.

 Below is a stepwise procedure for creating the palatal obturator (Figure 7).

The child was positioned upright with the face facing forward and a finger-loaded impression was made using elastomeric putty impression material.

The child was encouraged to suck during the procedure, which helped capture detailed tissue impressions and undercuts. Once the material had been set, it was gently removed from the child's mouth (Figure 7A). The impression was then poured with dental stone, and the master cast was created using the inversion method (Figures 7B, 7C). A single-visit feeding obturator was fabricated using clear, flexible thermal-forming material with an extraoral midline extension for a hole to secure a safety thread (Figure 7D).

The obturator was completed and polished to create smooth surfaces. Upon insertion, the child was able to comfortably suck with the feeding obturator in place. The mother was able to feed the child immediately (Figure 7E). This case underscores the significance of early, non-surgical interventions in addressing feeding difficulties in neonates with cleft palate, ensuring proper nutrition and reducing the risk of aspiration while awaiting definitive surgical correction.

## Discussion

A cleft palate disrupts the intraoral pressure necessary for suction and promotes the nasal reflux of food, which increases the risk of middle ear infections. Feeding becomes an immediate concern for newborns with CLP, highlighting the important role of the dentist and the use of a feeding plate [3]. A feeding appliance helps to close the gap between the oral and nasal cavities. In infants with CLP, a feeding obturator becomes essential for the infant's health, as surgical treatment typically begins at two to three months of age. Presurgical orthopedic appliance fabrication for children with clefts also starts with taking an impression [4]. However, there is limited literature available regarding the specific impression techniques required to create these maxillary appliances.

Various strategies have been suggested in the literature to address the challenges of feeding and development in infants with CLP, particularly in the early stages [4]. These methods aim to improve feeding efficiency and support growth before surgical intervention is possible. Some common approaches are listed below.

Specially designed nipples with wide openings

These nipples are designed to facilitate milk flow with minimal effort, reducing the need for sucking. The wider opening helps control the milk flow, making feeding easier for infants with CLP who may struggle with conventional breastfeeding or bottle-feeding [5].

Orogastric or nasogastric tubes

These feeding tubes provide a reliable method of delivering nutrition when oral feeding is difficult. However, their use is typically short-term, as there are risks of complications, and the goal is to transition to oral feeding as soon as possible [5].

Presurgical infant maxillary orthopedics (PIMO)

This approach involves the use of orthodontic devices prior to surgery to align and approximate the cleft segments. The goal is to improve the alignment of the jaw and palate, which may result in better surgical outcomes. The effectiveness of this method can vary depending on the severity of the cleft [5].

Surgical repair

Surgery is commonly used to close the cleft and improve functions such as feeding, speech, and overall oral development. However, it may not be effective in all cases, especially when there is a large gap between the cleft segments, requiring further interventions [5].

Palatal obturator

This prosthetic device covers the cleft in the palate, allowing for more efficient feeding by preventing milk from entering the nasal cavity. It can also assist in speech and language development by helping to close the palate during early speech attempts. Despite its benefits, its use in speech development is often underexplored, and further research is needed in this area [5].

Although these interventions can help in the early stages of managing CLP, their success and effectiveness vary depending on the individual case. More research is needed to better understand the long-term benefits, particularly in terms of speech and language development, an area that has received comparatively less attention in existing studies [5].

The impression procedure is essential for the fabrication of an obturator and should be performed in the presence of a pediatrician in the neonatal intensive care unit. Key factors to consider include patient positioning, tray selection, and choice of impression material. Several infant positioning techniques have been used for making CLP impressions, such as face down, upright, horizontally raised to a sitting position as the impression sets, or even inverted upside down. Various impression materials, including alginate, low-fusing impression compound, and elastomeric (rubber base) materials, are commonly used for neonates with CLP. However, alginate is not ideal due to its low tear strength, which can cause tearing during impression-making. Low-fusing impression compound, being a thermoplastic material, can sometimes cause burns, and its volatile components may pose a health hazard to the neonate [6].

In our experience, elastomeric materials are the most suitable for making CLP impressions as they offer several advantages, including high tear strength, good elastic properties, accurate reproduction of surface details, and long-term dimensional stability, which allows for multiple pours without complications. Various biomaterials for maxillofacial prosthetics, such as acrylic resins, visible light-cured acrylic, acrylic polymers, and silicones, are available, but no single material currently combines all the desired properties. Acrylic resin was chosen for the obturator because it is widely available, durable, and can be fabricated with thin margins, making it an optimal choice for this purpose [6].

## Conclusions

The nutritional demands of children with CLP are the same as unaffected children. A palatal obturator helps overcome the obstacles required to fulfill the nutritional demands of affected children and the psychological well-being of parents. The use of a feeding obturator and breastfeeding should be encouraged in isolated CLP cases. To ensure its proper usage, frequent follow-up and counseling sessions should be done with the parents. The feeding appliance usage and acceptance are made easier by the elastomeric material's resilience, which easily conforms to the curves of the child's mouth. The device is also secure and very well retained regardless of the pressures applied to it during the feeding session. The demands of both mother and child may be successfully met by the utilization of a feeding appliance, a bottle, or breastfeeding and proper lactation awareness. In conclusion, this article outlines a simple and low-risk procedure for fabricating a palatal obturator for neonates. The collaborative team approach is crucial to achieving success in the care of these patients. A palatal obturator can play a significant role in alleviating feeding difficulties and supporting speech and language development in CLP patients.
